# A meta-analysis and trial sequential analysis of randomised controlled trials comparing nonoperative and operative management of chest trauma with multiple rib fractures

**DOI:** 10.1186/s13017-024-00540-z

**Published:** 2024-03-19

**Authors:** Ryo Hisamune, Mako Kobayashi, Karin Nakasato, Taiga Yamazaki, Noritaka Ushio, Katsunori Mochizuki, Akira Takasu, Kazuma Yamakawa

**Affiliations:** 1 Department of Emergency and Critical Care Medicine, Osaka Medical and Pharmaceutical University, 2-7 Daigakumachi, Takatsuki, Osaka 569-8686 Japan; 2 Faculty of Medicine, Osaka Medical and Pharmaceutical University, 2-7 Daigakumachi, Takatsuki, Osaka 569-8686 Japan

**Keywords:** Chest trauma, Flail chest, Fracture stabilization, Rib fractures, Surgery fixation, Thoracic injury

## Abstract

**Background:**

Operative treatment of traumatic rib fractures for better outcomes remains under debate. Surgical stabilization of rib fractures has dramatically increased in the last decade. This study aimed to perform a systematic review and meta-analysis of randomised controlled trials (RCTs) to assess the effectiveness and safety of operative treatment compared to conservative treatment in adult patients with traumatic multiple rib fractures.

**Methods:**

A systematic literature review was performed according to the preferred reporting items for systematic reviews and meta-analyses guidelines. We searched MEDLINE, Scopus, and Cochrane Central Register of Controlled Trials and used the Cochrane Risk-of-Bias 2 tool to evaluate methodological quality. Relative risks with 95% confidence interval (CI) were calculated for outcomes: all-cause mortality, pneumonia incidence, and number of mechanical ventilation days. Overall certainty of evidence was evaluated with the Grading of Recommendations Assessment, Development and Evaluation (GRADE) approach, with trial sequential analysis performed to establish implications for further research.

**Results:**

From 719 records, we included nine RCTs, which recruited 862 patients. Patients were assigned to the operative group (received surgical stabilization of chest wall injury, n = 423) or control group (n = 439). All-cause mortality was not significantly different (RR = 0.53; 95% CI 0.21 to 1.38, *P* = 0.35, *I*^*2*^ = 11%) between the two groups. However, in the operative group, duration of mechanical ventilation (mean difference -4.62; 95% CI -7.64 to -1.60, *P* < 0.00001, *I*^*2*^ = 94%) and length of intensive care unit stay (mean difference -3.05; 95% CI -5.87 to -0.22; *P* < 0.00001, *I*^*2*^ = 96%) were significantly shorter, and pneumonia incidence (RR = 0.57; 95% CI 0.35 to 0.92; *P* = 0.02, *I*^*2*^ = 57%) was significantly lower. Trial sequential analysis for mortality indicated insufficient sample size for a definitive judgment. GRADE showed this meta-analysis to have very low to low confidence.

**Conclusion:**

Meta-analysis of large-scale trials showed that surgical stabilization of multiple rib fractures shortened the duration of mechanical ventilation and reduced the incidence of pneumonia but lacked clear evidence for improvement of mortality compared to conservative treatment. Trial sequential analysis suggested the need for more cases, and GRADE highlighted low certainty, emphasizing the necessity for further targeted RCTs, especially in mechanically ventilated patients.

*Systematic review registration*: UMIN Clinical Trials Registry UMIN000049365.

**Supplementary Information:**

The online version contains supplementary material available at 10.1186/s13017-024-00540-z.

## Background

Traumatic deaths account for approximately 10% of all deaths worldwide [1]. Especially, chest trauma comprises up to 25% of the annual trauma cases [2], and rib fractures are the most common type of blunt trauma, accounting for 43% [3]. The common causes of trauma include road traffic accidents, high falls, crush forces, and direct violence. Rib fractures resulting from these causes can range from simple to a flail segment [4]. One of the characteristic clinical symptoms is a flail chest, which is defined as three or more sequential rib fractures at more than one site that result in paradoxical movement of the chest wall, alteration of respiratory mechanics, and, frequently, respiratory failure. Generally, it has been difficult to control pain in these injuries, which show a high incidence of complications, including chest wall instability, severe pulmonary restriction owing to paradoxical movement of the flail segment, and loss of lung volume [5]. The combination of chest wall instability, decreased lung capacity, and pain can result in decreased lung function and the need for prolonged ventilation.

Prolonged mechanical ventilation is associated with high rates of pneumonia, tracheostomy, barotrauma, long-term intensive care unit (ICU) stays, and high medical costs [6]. Rib fractures also represent a significant loss of working days and may reduce a patient’s quality of life for several months after injury [7]. The treatment strategy for rib fractures can be divided into two main categories: conservative treatment and surgical treatment. Although conservative treatment used to be the main strategy, surgical treatment to stabilize rib fractures (SSRF) has dramatically increased in the last decade [8], with rates of SSRF having risen from less than 1% to over 10% [9]. Conceptually, SSRF applies the basic orthopaedic principles of reduction and fixation to rib fractures, restoring chest wall stability, relieving pain, and improving respiratory failure. The most prevalent treatment of severe chest wall injuries consists of non-operative management via intubation and intermittent positive-pressure ventilation as needed, analgesia, pulmonary toilet, chest tube drainage, and chest physiotherapy. Recently, retrospective studies and multiple randomised controlled trials (RCTs) have reported that surgical treatment improved clinical outcomes compared to conservative treatment.

As mentioned above, the optimal strategy for traumatic multiple rib fractures is controversial due to limited available evidence. However, novel large-scale RCTs performed by Dehghan et al. [10] and Meyer et al. [11] were recently published. Here, we describe our systematic review and meta-analysis of RCTs performed to assess the certainty of evidence for determining the optimal strategy for patients with traumatic rib fractures.

## Methods and designs

### Protocol registration

This study protocol was registered in the University Hospital Medical Information Network (UMIN) Clinical Trials Registry (https://www.umin.ac.jp/ctr/index-j.htm) (Registration No. UMIN000049365). The protocol follows the Preferred Reporting Items for Systematic Reviews and Meta-Analyses Protocols (PRISMA-P) statements [12], and this systematic review and meta-analysis followed the PRISMA statement [13].

### Focused review questions

The purpose of this review was to explore the effectiveness and safety of operative management for traumatic chest wall injury.

### Types of studies

We included only RCTs that compared operative management in adults with multiple rib fractures to non-operative management. We excluded the following article types: cohort studies, case–control studies, experimental animal studies, narrative reviews, correspondence, case reports, expert opinions, and editorials from the study.

### Participants

We included RCTs that enrolled adult patients aged 16 years or older suffering traumatic rib fractures with or without flail chest in which three or more adjacent ribs were fractured in at least two places and who required surgery.

### Interventions and comparators

We included RCTs that describe an operative management group versus a non-operative management group for traumatic multiple rib fractures. Studies were included if surgery was performed within one week of patient enrolment. The number of rib fractures requiring repair varied across the studies, and rib fracture stabilization was primarily achieved using screws and plates, and wires. Also, the choice of surgical specialists, such as trauma surgeons, orthopaedic surgeons, or thoracic surgeons, depended on the studies. All patients in each group received standard-of-care treatment, including mechanical ventilation as needed, pain control management, chest drainage as needed, antibiotics administration, and pulmonary physiotherapy.

### Outcome assessments

The primary outcome parameter was all-cause mortality. The secondary outcome parameters of duration of mechanical ventilation, incidence of pneumonia, length of ICU stay, length of hospital stay, and need for tracheostomy were also assessed.

### Search strategy

We searched the following databases for relevant studies: MEDLINE (via PubMed), Scopus, and the Cochrane Central Register of Controlled Trials. We developed a search strategy in MEDLINE using a combination of keywords and Medical Subject Headings (MeSH) terms as listed in Additional file 4. We also screened the reference lists of all relevant papers for additional studies. Our MEDLINE search strategy was adapted appropriately for searches in the other two databases. No language or time restrictions were applied to the electronic searches. Our systematic search was conducted in September 2023.

### Citation management and screening

Citations were stored, and duplicates were removed using EndNote software ver. 20.4 (Thomson Reuters, Toronto, Ontario, Canada). Studies were screened initially according to title and abstract by two authors (KN and TY) independently, and those not meeting the criteria were discarded. Disagreements were resolved by discussion and referral to a third author (KY) if necessary. After this initial stage, the full text of all remaining studies was reviewed, and disagreements were resolved in the same way as in the initial screening. We used the Rayyan QCRI website (http://rayyan.qcri.org) [14] in this screening process. We documented the study selection process in a PRISMA flow diagram.

### Data extraction

Two authors (KN and TY) independently extracted the study characteristics from each included study and transferred the information into a study-specific format. The following information was extracted from the included studies: the number of patients in the intervention and control groups, type of surgery, number of patients who were on mechanical ventilation at the time of enrolment, number of fractured ribs, reported outcomes, and the respective mean and standard deviation or frequency data. Outcome data were double-checked, consolidated, and included in the meta-analysis software. Adjudication by a third author (KY) was used if necessary.

### Assessment of risk of bias within studies

Independent reviewers (KN, TY, and RH) assessed the risk of bias in the individual trials as the methodological quality of the articles. Disagreements were resolved by discussion and consultation with a third author (KY). To evaluate the risk of bias in the individual RCTs, we followed the recommendations of the Cochrane Handbook for Systematic Reviews of Interventions and used the Cochrane Collaboration’s tool for assessing the risk of bias in randomised trials (Rob 2) [15]. For each domain, we assigned a judgment regarding the risk of bias as “high risk”, “low risk”, or “some concerns”. We attempted to contact a trial’s corresponding author for clarification when insufficient detail was reported to assess the risk of bias.

### Statistical analysis

Statistical analyses were performed using Review Manager 5.4 software (Nordic Cochrane Centre, Cochrane Collaboration) and R software version 4.2.3. For each included trial, we calculated the relative risk with 95% confidence interval (CI) for all outcome measures. Continuous variables are reported as a mean difference (MD) with 95% CI using a random effects inverse variance model. Dichotomous variables were analysed using the Mantel–Haenszel model and were reported as a risk ratio (RR) with the random effects model.

### Trial sequential analysis

Trial sequential analysis (TSA) was performed using Trial Sequential Analysis software ver. 0.9.5.10 beta [Computer program] (The Copenhagen Trial Unit, Centre for Clinical Intervention Research, The Capital Region, Copenhagen University Hospital-Rigshospitalet 2021). We assessed the adequacy of the available number of patients and performed TSA to establish the implication for further research. We calculated the required diversity-adjusted information size. Diversity, which indicates the percentage of variability between trials, is calculated as the sum of the between-trial variability and a sampling error estimate based on the required information size. We performed TSA with the goal of maintaining an overall 5% risk of type I error, which is considered the standard for most meta-analyses and systematic reviews. The required information size was also calculated (α error of 5%, β error of 20%) [16, 17]. In theory, firm evidence is likely established if the trial sequential monitoring boundary is crossed before reaching the required information size, but if this boundary is not crossed, it is highly likely that trials will need to be continued.

### Assessment of heterogeneity

We investigated heterogeneity initially by visual examination of forest plots. Statistical heterogeneity was evaluated informally from forest plots of the study estimates and more formally using the *χ*^*2*^ test (*P*-value < 0.1 = significant heterogeneity) and *I*^*2*^ statistic (*I*^*2*^ > 50% = significant high heterogeneity). We performed sensitivity analyses using low-risk-of-bias trials and trials of flail segments of rib fractures.

### Assessment of publication biases

Funnel plots were conducted to evaluate potential publication bias in the current literature. We did not conduct a quantitative assessment of small studies or evaluate the presence of publication bias due to the inclusion of fewer than 10 studies in this meta-analysis [18].

### Rating certainty of evidence with the grading of recommendations assessment, development, and evaluation (GRADE) approach

The risks of systematic errors (bias) and random errors were assessed, and the overall certainty of evidence was evaluated using the GRADE guidelines [19]. We labeled studies as having very low-, low-, moderate-, or high-quality evidence based on the presence of risk of bias, inconsistency of results, imprecision, publication bias, and magnitude of treatment effects.

## Results

### Study identification and selection

**Fig. 1:**
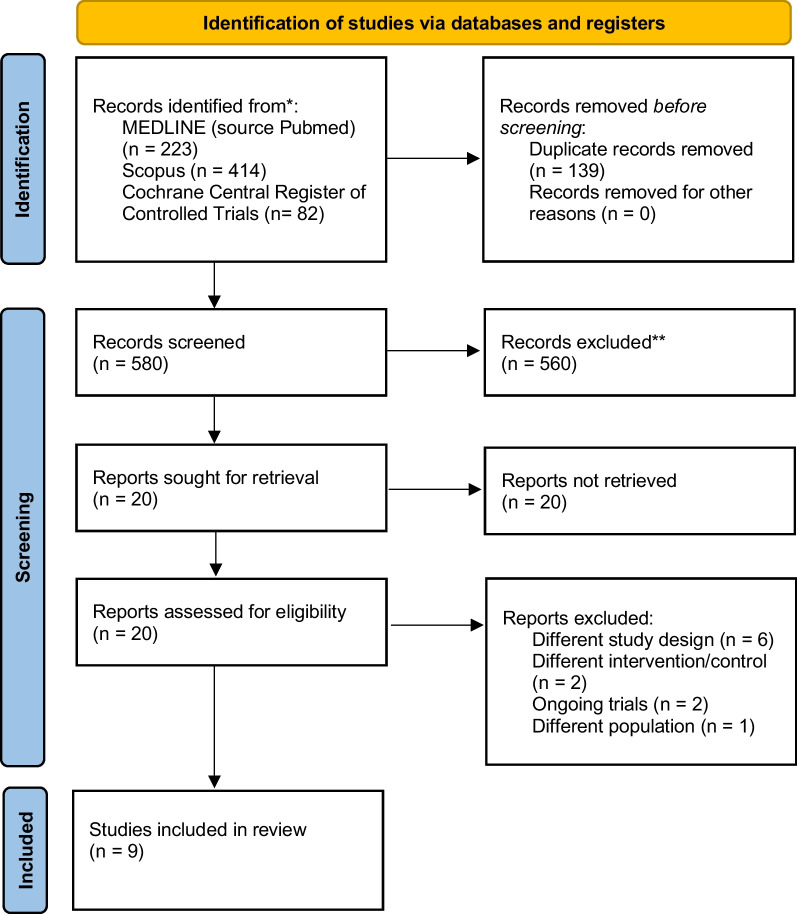
PRISMA flow chart of study screening and selection. The search strategy in MEDLINE, Scopus, and Cochrane Central Registry of Controlled study yielded 580 studies. We reviewed 20 studies for inclusion and exclusion criteria and finally included 9 studies in the meta-analysis

In total, 719 articles were retrieved, including 223 from PubMed, 414 from Scopus, and 82 from Cochrane Central Register of Controlled Trials by the search strategy, as shown in Fig. 1. Excluding duplicates, 580 articles underwent primary screening and 20 underwent secondary screening. Ultimately, 9 articles were included in the final analysis [10, 11, 20–26]. Of the total of 862 patients included in the articles under review, 423 were treated with operative treatment, and 439 were treated with conservation treatment. Seven RCTs [10, 20–24, 26] involved patients with flail segment and clinical flail chest, one study [11] involved patients with flail segment but no clinical flail chest, one study [25] involved patients with simple rib fractures without clinical or radiological flail chest, and one study included only men [23]. The characteristics of the included studies are summarised in Table 1.

**Table 1:** Characteristics of the included studies

Author	Year	Fracture type	Primary outcome	Number of patients			Age (mean)		Number of males		Number of fractures		ISS (mean)		MV at randomization (%)	
				SM	NSM	Total	SM	NSM	SM	NSM	SM	NSM	SM	NSM	SM	NSM
Tanaka [20]	2002	Flail	MV days	18	19	37	43	46	43	46	8.2	8.2	33	30	100	100
Granetzny [21]	2005	Flail	Pulmonary function	20	20	40	40.5	36	17	14	4.4	4.6	16.8	18	35	45
Marasco [22]	2012	Flail	MV days/ ICU stay	23	23	46	57.8	59.3	20	20	11	11.3	35	30	100	100
Wu [23]	2015	Flail	MV days/ ICU stay	75	89	164	52	51	75	89	8.7	8.3	20.3	18.5	28	30.3
Liu [24]	2019	Flail	Mortality	27	26	53	41.6	39.6	21	20	6.3	5	29	27.7	NA	NA
Pieracci [25]	2020	Simple	Pain	51	59	110	54.6	55.3	39	43	7	7	13	14	0	0
Marasco [26]	2022	Flail	Pain	61	63	124	59.1	55	48	51	6.7	6.7	14.7	16	0	0
Dehghan [10]	2022	Flail or severe chest wall deformity	Ventilator-free days	108	99	207	52.9	53.2	81	75	10.1	10.5	25.3	26	41	46
Meyer [11]	2023	Flail segment without clinical flail chest	Length of hospital stay	42	42	84	50	49	28	31	8.3	7.7	23.7	21.3	NA	NA

*ISS* Injury Severity Score; *MV* mechanical ventilation; *SM* surgical management; *NSM* non-surgical management; *ICU* intensive care unit; *NA* not available

### Assessment of bias

The identified studies exhibited heterogeneity in their inclusion criteria, surgical techniques, time to surgery, definition of flail chest, assessed outcomes, and length of follow-up. Therefore, we used random effect models for the analysis. The risk of individual within-study bias is represented in the Rob 2 traffic-light diagram shown in Additional file 1: Fig. S1. All of the Rob 2 assessment domains excluding the measurement of outcome domain had at least one or some concerns. One RCT raised high risk in its deviation from the intended intervention domain. Two RCTs were assigned a low risk of bias.

### Outcomes

#### Mortality

**Fig. 2:**
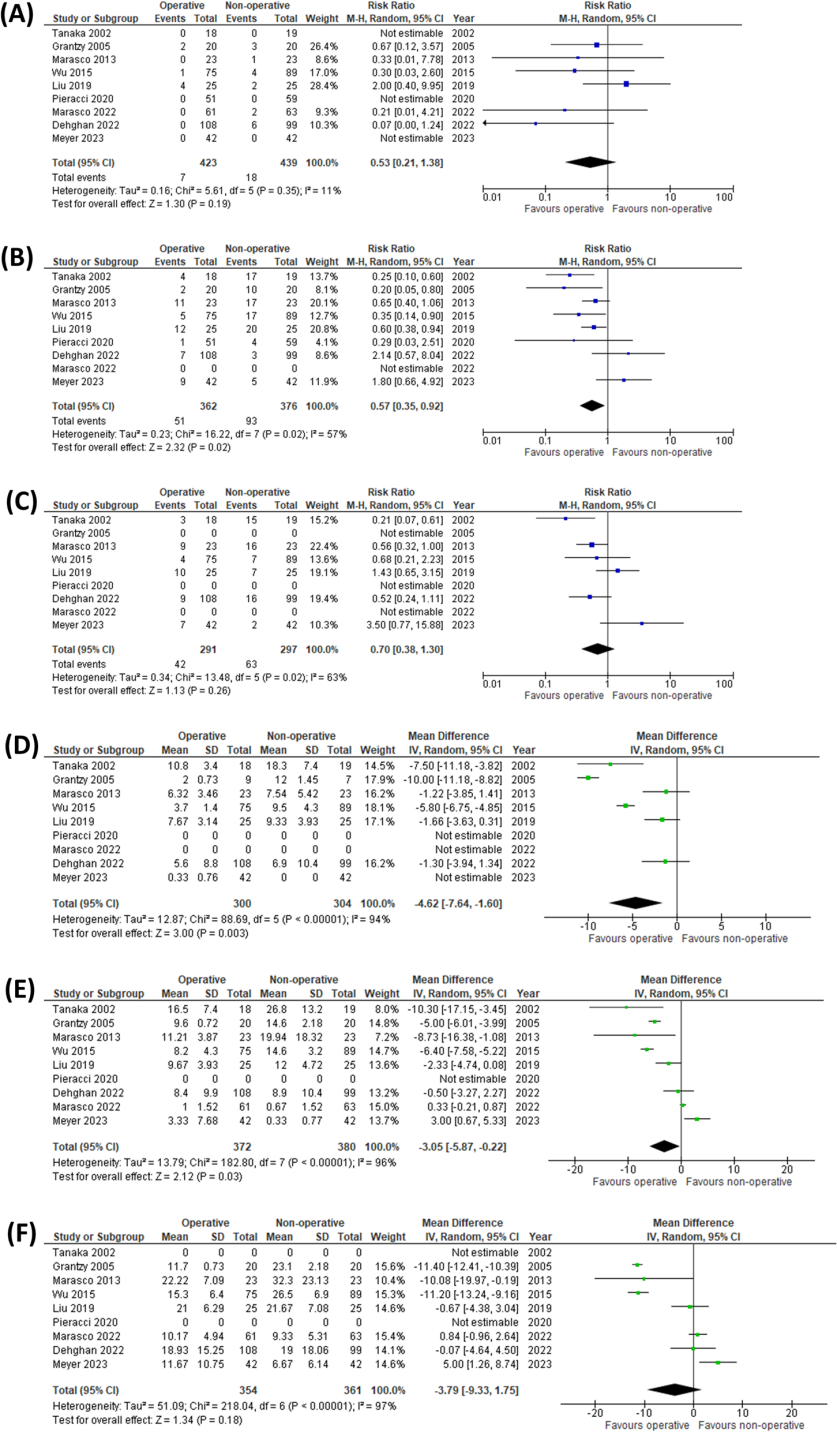
Forest plot of studies for operative vs non-operative management. Forest plots of studies examining **A** mortality, **B** the incidence of pneumonia, **C** the need for tracheostomy, **D** the duration of echanical ventilation, **E** the length of ICU stay, and **F** the length of hospital stay for operative vs non-operative management. M-H, Mantel–Haenszel; CI, confidence interval; and SD, standard deviation; IV, inverse variance

All of the studies reported mortality. Complete data were extracted from all studies, with no statistical heterogeneity noted between groups *(I*^*2*^ = 11%, *P* = 0.35). There was no significant difference in the mortality rate between the groups (RR = 0.53; 95% CI 0.21 to 1.38; *P* = 0.19) (Fig. 2A).

#### Incidence of pneumonia

Complete data were shown in 8 studies [10, 11, 20–25], with statistical heterogeneity noted between groups (*I*^*2*^ = 57%, *P* = 0.02). A combined 144 events occurred in the 738 patients. There was a significant difference favouring the operative group (RR = 0.57; 95% CI 0.35 to 0.92; *P* = 0.02) (Fig. 2B).

#### Need for tracheostomy

Complete data were shown in 6 studies [10, 11, 20, 22–24], with statistical heterogeneity noted between groups (*I*^*2*^ = 63%, *P* = 0.02). A combined 105 events occurred in the 588 patients. There was no significant difference between the groups (RR = 0.70; 95% CI 0.38 to 1.30; *P* = 0.26) (Fig. 2C).

#### Duration of mechanical ventilation

Complete data were shown in 7 studies [10, 11, 20–24], with statistical heterogeneity noted between groups (*I*^*2*^ = 94%,* P* < 0.001). There was a significant difference favouring the operative group (MD -4.62; 95% CI -7.64 to -1.60; *P* = 0.003) (Fig. 2D).

#### ICU length of stay

Complete data were shown in 8 studies [10, 11, 20–24, 26], with statistical heterogeneity noted between groups (*I*^*2*^ = 96%,* P* < 0.01). There was a significant difference favouring the operative group (MD -3.05; 95% CI -5.87 to -0.22; *P* = 0.03) (Fig. 2E).

#### Hospital length of stay

Complete data were shown in 7 studies [10, 11, 21–24, 26], with statistical heterogeneity noted between groups (*I*^*2*^ = 97%, *P* < 0.001). There was no significant difference between the groups (MD -3.79; 95% CI -9.33 to 1.75; *P* = 0.18) (Fig. 2F).

### Trial sequential analysis

**Fig. 3:**
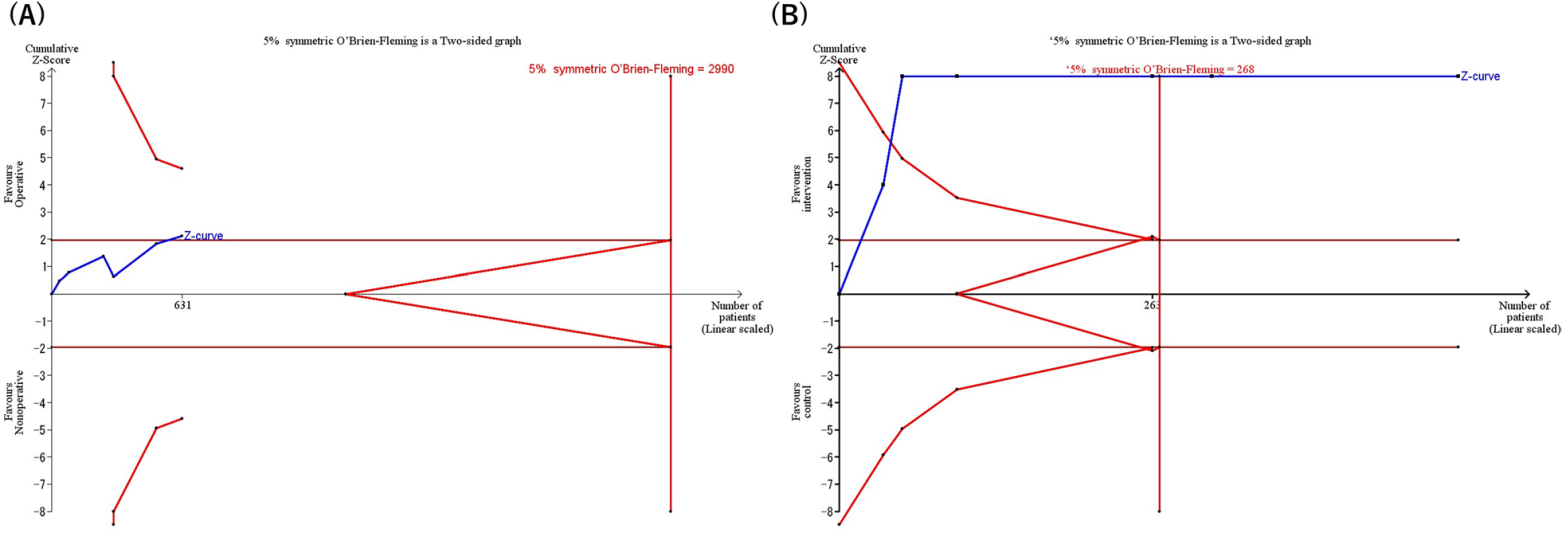
Trial sequential analysis results. **A** Trial sequential analysis for mortality. **B** Trial sequential analysis for duration of mechanical ventilation

We performed TSA for mortality after adjusting for the information size considering the presence of heterogeneity and used data from all 9 RCTs involving 762 patients. The calculated information size, adjusted for required diversity, and targeting a 5% risk of type I error, 20% risk of type II error, and low risk of bias trials investigating the expected effect (relative risk reduction [RRR] of 30%), amounted to 2290 patients. The cumulative Z curve only intersected the futility boundaries but did not intersect the monitoring boundary, indicating an insufficient number of studies to show substantial evidence for a -30% RRR in mortality with operative treatment (Fig. 3A). Given an accumulated information size of 2290 patients and no monitoring boundary crossings up to this point, we currently possess only 33.3% of the necessary information size required to make a definitive judgment on accepting or rejecting a 30% RRR for all-cause mortality.

Additionally, we performed TSA for the secondary outcome of the duration of mechanical ventilation, utilizing data from 7 of the RCTs involving 520 patients. The cumulative Z curve intersected both the futility boundaries and the monitoring boundary before reaching the information size, indicating firm evidence of the effect of operative treatment on the duration of mechanical ventilation compared to conservative treatment (Fig. 3B).

### Quality of evidence

Very low or low confidence was assigned to this meta-analysis of the primary outcomes. This suggested that the actual effect might differ from the estimated effect due to severe risk of bias and issues of imprecision that could potentially introduce bias into the meta-analysis. The quality assessment is detailed in Table 2. No publication bias was apparent after inspection of the funnel plots of the primary outcomes.

**Table 2:** Assessment sheet of overall quality of evidence by Grading of Recommendation, Assessment, Development, and Evaluations (GRADE) guidelines

Certainty assessment							No of patients		Effect		Certainty	Importance
No of studies	Study design	Risk of bias	Inconsistency	Indirectness	Imprecision	Other considerations	Surgery	Control	Relative (95% CI)	Absolute (95% CI)		
*All-cause mortality*												
9	Randomised trials	Serious^a^	Not serious	Not serious	Serious^b^	Publication bias strongly suspected^c^	7/423 (1.7%)	18/439 (4.1%)	RR 0.53 (0.21 to 1.38)	19 fewer per 1,000 (from 32 fewer to 16 more)	⨁◯◯◯ Very low	
*Pneumonia*												
8	Randomised trials	Serious^a^	Not serious	Not serious	Not serious	None	51/362 (14.1%)	93/376 (24.7%)	RR 0.57 (0.35 to 0.92)	106 fewer per 1,000 (from 161 to 20 fewer)	⨁⨁⨁◯ Moderate	
*Tracheostomy*												
6	Randomised trials	Serious^a^	Not serious	Not serious	Serious^b,d^	None	42/291 (14.4%)	63/297 (21.2%)	RR 0.70 (0.38 to 1.30)	64 fewer per 1,000 (from 132 fewer to 64 more)	⨁⨁◯◯ Low	
*Length of ICU stay*												
8	Randomised trials	Serious^a^	Serious^e^	Not serious	Not serious	Publication bias strongly suspected^c^	372	380	–	MD 3.05 days lower (5.87 lower to 0.22 lower)	⨁◯◯◯ Very low	
*Length of hospital stay*												
7	Randomised trials	Serious^a^	Serious^e^	Not serious	Serious^d^	None	354	361	–	MD 3.79 days lower (9.33 lower to 1.75 higher)	⨁◯◯◯ Very low	
*Duration of mechanical ventilation*												
7	Randomised trials	Serious^a^	Serious^e^	Not serious	Not serious	None	300	304	–	MD 4.62 days lower (7.64 lower to 1.6 lower)	⨁⨁◯◯ Low	

^a^Seven randomised clinical trials had some concerns of bias

^b^Insufficient optimal information size

^c^Funnel plot indicates evidence of significant publication bias

^d^Large confidence interval

^e^High heterogeneity (I2 > 75%)

CI, confidence interval; RR, risk ratio; ICU, intensive care unit; MD, mean difference

### Sensitivity analysis

Results of the sensitivity analysis of outcomes using the low risk of bias studies, excluding Marasco et al. [26], are presented in Additional file 2: Fig. S2, and those of the outcomes using the studies of the flail segments of rib fractures, excluding Pieracci et al. [25], are presented in Additional file 3: Fig. S3. The results of these analyses were consistent with the main results.

## Discussion

### Summary of results

Our review of 9 RCTs including more than 800 patients thoroughly assessed the effectiveness and safety of operative management for multiple rib fractures compared to conservative treatment. Our meta-analysis revealed that patients who underwent surgical procedures for multiple rib fractures gained significant clinical benefits, including faster weaning from the mechanical ventilator, a reduced incidence of pneumonia, and shorter ICU stays, compared to those receiving conservative treatment. However, there were no differences in mortality, length of hospital stay, and need for tracheostomy between the groups. Our meta-analysis had, to our best knowledge, the unique characteristics of having the largest sample size to date on this topic and being the first study to assess these data by TSA.

### Mortality

The effectiveness of surgical management began to be reported about 30 years ago [27]. The first RCT on surgical therapy for multiple rib fractures was conducted by Tanaka et al. [20] in 2002. Since then, many RCTs have been reported in recent years [10, 11, 21–26]. Most of them have found no difference in mortality but have reported significant differences in the improvement of clinical outcomes related to complications and the use of mechanical ventilation. In cases of thoracic trauma, pneumonia stands out as a leading cause of death. Previous RCTs predating the study conducted by Dehghan et al. [10] reported a significant reduction in the incidence of pneumonia in the surgical intervention group compared to the conservative group. This observation was consistent with the findings of various systematic reviews and meta-analyses [9, 28–30]. However, the recent RCTs by Dehghan et al. [10] and Meyer et al. [11], conducted over the past 2–3 years, did not show a significant difference in the incidence of pneumonia between the groups. Notably, the non-operative treatment groups in these recent RCTs were treated according to standardised protocols, including those for analgesia with epidural anaesthesia, comprehensive management, and weaning procedures. It is plausible that these modern, well-established protocols may have contributed to the discrepant results. Our TSA results regarding mortality did not show significant efficacy, likely due to the insufficient sample size.

### Duration of mechanical ventilation

Prolonged mechanical ventilation exposes patients to well-documented risks, including an elevated susceptibility to pneumonia, sepsis, weakened respiratory muscles, and the necessity for tracheostomy [31, 32]. Therefore, it is important to reduce the number of days of mechanical ventilation as part of the strategy for treating multiple rib fractures. The early surgical procedure restores chest wall integrity and prevents the development of permanently damaging sequelae [21] but carries with it potential complications such as wound infection, fixation failure, and emphysema. However, when comparing prolonged mechanical ventilation and surgical procedures, the likelihood of risks associated with prolonged mechanical ventilation is higher than that with surgical treatment [33]. Our results showed a significantly shorter duration of mechanical ventilation in the surgical treatment group than in the conservative treatment group. However, the heterogeneity of the results was large and the results varied, making them unreliable. Other than the Dehghan et al. study [10], all five of these studies were conducted at a single institution, had small numbers of patients (less than 80 patients), and two of the studies used outdated surgical fixation methods. The Dehghan et al. study [10] is the most recent and has the largest number of patients and institutions, making its findings credible. However, their study found a benefit from surgical treatment for patients already on mechanical ventilation at study enrolment. The observed benefit in this patient group aligns with the findings of two other RCTs [20, 24]. All patients in these RCTs were initially on mechanical ventilation before their inclusion in the studies. Surgical treatment for multiple rib fractures may have potential benefits, particularly for patients needing mechanical ventilation on admission. Our TSA results regarding the duration of mechanical ventilation showed that enough evidence exists to indicate a benefit from surgical treatment. These results suggest that surgical treatment may be more effective particularly for ventilator-dependent patients with multiple rib fractures than for non-ventilator-dependent patients.

### Limitations

There are several potential limitations in this systematic review. First, in the RCTs of surgical treatment, the patients, evaluators, and medical staff involved in the patients’ care were loosely blinded to the study groups, thus potentially introducing bias in the outcomes. Second, there were no uniform criteria for identifying the outcomes, such as diagnostic criteria for pneumonia, weaning criteria for mechanical ventilation, and timing of discharge or transfer from the ICU. This raised concerns about measurement of bias in our analysis. Third, surgical procedure methods (fixation method and number of fixations) and surgical departments performing the surgery differed among the studies. There is also a 20-year period between the first RCT and the most recent RCT in the present analysis. During this time, there have been changes in surgical technique and local analgesia and advances in rib fixation devices that have led to shorter operative times.

## Conclusion

Although this meta-analysis including large-scale RCTs demonstrated a reduction in the duration of mechanical ventilation and the incidence of pneumonia, improvement in mortality rates was challenging to establish. TSA indicated the need for a larger number of cases, and the low certainty observed in the GRADE system assessment highlights the necessity for more focused RCTs, particularly among patients requiring mechanical ventilation.

### Supplementary Information

**Additional file 1: Fig. S1.** Traffic light diagram illustrating the critical evaluation of randomised clinical trials using the Cochrane Collaboration’s Risk of Bias Tool for Randomized Trials (RoB 2). Green traffic light indicates low risk; yellow light, some concerns; and red light, high risk.

**Additional file 2: Fig. S2. **Sensitivity analysis for outcomes using studies with low risk of bias. Forest plots of studies examining (A) mortality, (B) the incidence of pneumonia, (C) the need for tracheostomy, (D) the duration of mechanical ventilation, (E) the length of ICU stay, and (F) the length of hospital stay for operative vs non-operative management. M-H, Mantel-Haenszel; CI, confidence interval; and SD, standard deviation; IV, inverse variance.

**Additional file 3: Fig. S3.** Sensitivity analysis for outcomes using studies of the flail segment of rib fractures. Forest plots of studies examining (A) mortality, (B) the incidence of pneumonia, (C) the need for tracheostomy, (D) the duration of mechanical ventilation, (E) the length of ICU stay, and (F) the length of hospital stay for operative vs non-operative management. M-H, Mantel-Haenszel; CI, confidence interval; and SD, standard deviation; IV, inverse variance.

**Additional file 4**. Search strategy in MEDLINE using a combination of keywords and Medical Subject Headings (MeSH) terms.

## Data Availability

The datasets analysed during the current study are available from the corresponding author upon reasonable request.

## Abbreviations

RCTs: Randomised controlled trials
ICU: Intensive care unit
SSRF: Surgical stabilization of rib fractures
UMIN: University Hospital Medical Information Network
PRISMA-P: Preferred Reporting Items for Systematic Reviews and Meta-Analyses Protocols
RRR: Relative risk reduction
TSA: Trial sequential analysis
