# Supplementary figures and images for: A meta-analysis and trial sequential analysis of randomised controlled trials comparing nonoperative and operative management of chest trauma with multiple rib fractures
Source: World J Emerg Surg. 2024 Mar 19;19:11. doi: 10.1186/s13017-024-00540-z (PMC10949653; doi:10.1186/s13017-024-00540-z)

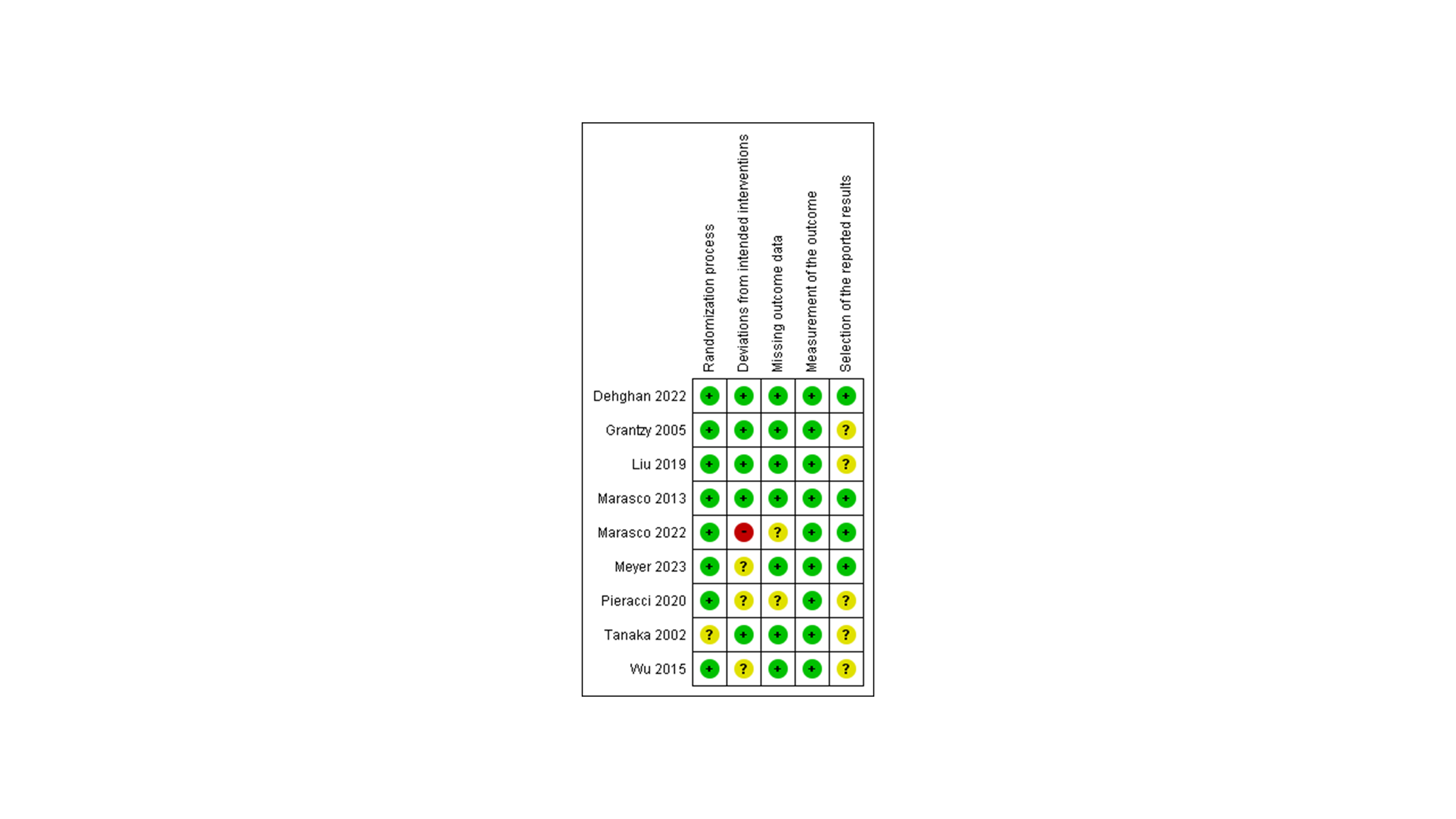

Supplement: Supplementary file 1 — Additional file 1: Fig. S1. Traffic light diagram illustrating the critical evaluation of randomised clinical trials using the Cochrane Collaboration’s Risk of Bias Tool for Randomized Trials (RoB 2). Green traffic light indicates low risk; yellow light, some concerns; and red light, high risk. [file 13017_2024_540_MOESM1_ESM.tif]

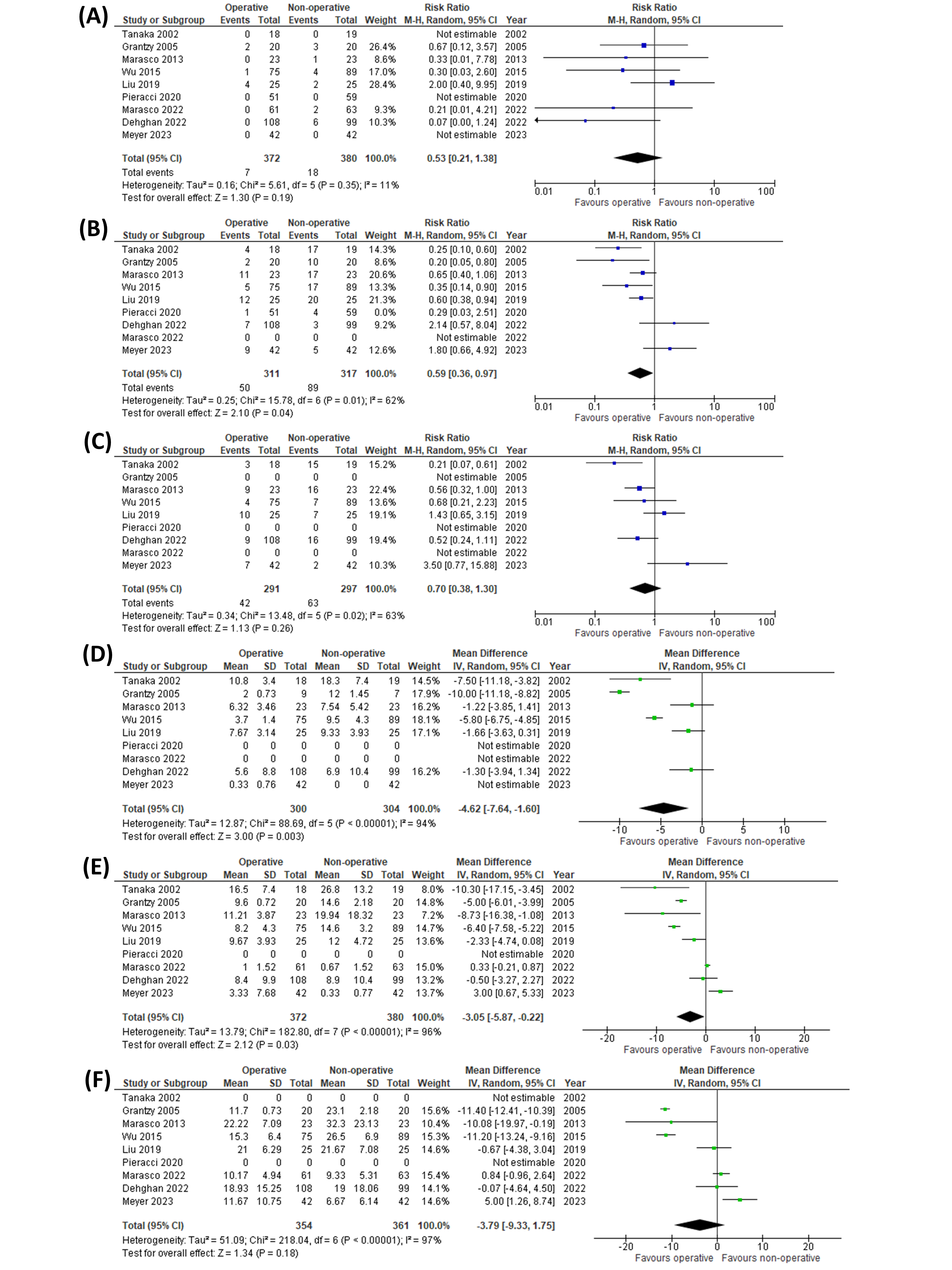

Supplement: Supplementary file 2 — Additional file 2: Fig. S2. Sensitivity analysis for outcomes using studies with low risk of bias. Forest plots of studies examining (A) mortality, (B) the incidence of pneumonia, (C) the need for tracheostomy, (D) the duration of mechanical ventilation, (E) the length of ICU stay, and (F) the length of hospital stay for operative vs non-operative management. M-H, Mantel-Haenszel; CI, confidence interval; and SD, standard deviation; IV, inverse variance. [file 13017_2024_540_MOESM2_ESM.tif]

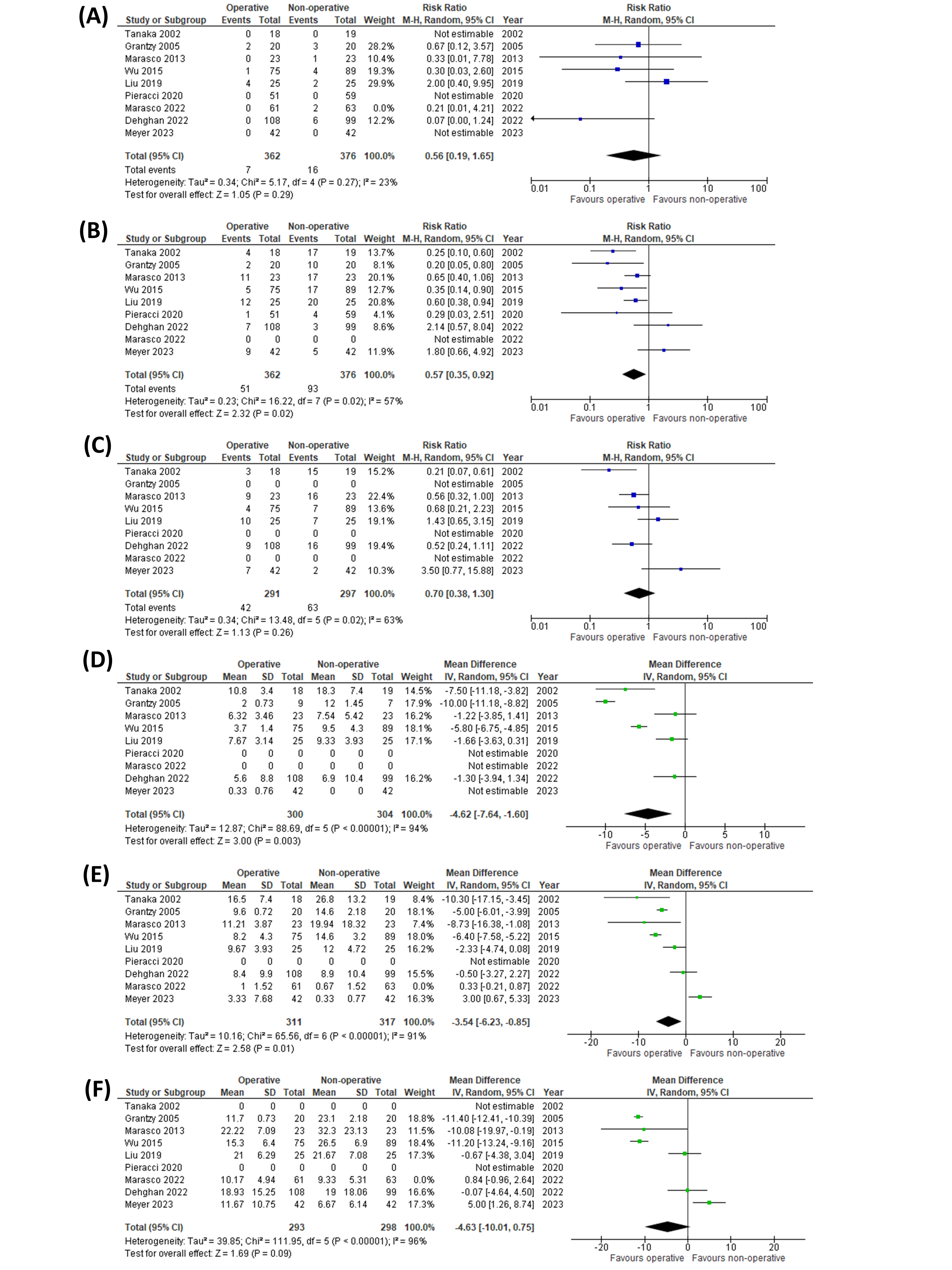

Supplement: Supplementary file 3 — Additional file 3: Fig. S3. Sensitivity analysis for outcomes using studies of the flail segment of rib fractures. Forest plots of studies examining (A) mortality, (B) the incidence of pneumonia, (C) the need for tracheostomy, (D) the duration of mechanical ventilation, (E) the length of ICU stay, and (F) the length of hospital stay for operative vs non-operative management. M-H, Mantel-Haenszel; CI, confidence interval; and SD, standard deviation; IV, inverse variance. [file 13017_2024_540_MOESM3_ESM.tif]
