# Supplementary material for: A meta-analysis and trial sequential analysis of randomised controlled trials comparing nonoperative and operative management of chest trauma with multiple rib fractures
Source: World J Emerg Surg. 2024 Mar 19;19:11. doi: 10.1186/s13017-024-00540-z (PMC10949653; doi:10.1186/s13017-024-00540-z)
Supplement: Supplementary file 4 — Additional file 4. Search strategy in MEDLINE using a combination of keywords and Medical Subject Headings (MeSH) terms. [file 13017_2024_540_MOESM4_ESM.docx]

***Supplementary materials***

***Search strategy***

We developed a search strategy in MEDLINE using a combination of keywords and Medical Subject Headings (MeSH) terms as follows: (“rib fractures”[MeSH Terms] OR rib fractures[Title/Abstract] OR “flail chest”[MeSH Terms] OR “flail chest”[Title/Abstract]) AND (“thoracic surgical procedures”[MeSH Terms] OR thoracic surgical procedures[Title/Abstract] OR (“fracture fixation”[MeSH Terms]) OR (fracture fixation[Title/Abstract]) OR “surgical procedure, operative”[MeSH Terms] or “surgical procedure, operative”[Title/Abstract] OR surgical[Title/Abstract]) AND (randomized controlled trial[Publication Type] OR controlled clinical trial[Publication Type] OR randomized[Title/Abstract] OR placebo[Title/Abstract] OR drug therapy[MeSH Subheadings] OR randomly[Title/Abstract] OR trial[Title/Abstract] OR groups[Title/Abstract] NOT (animals[MeSH Terms] NOT humans[MeSH Terms])).
